# German Health Update (GEDA 2019/2020-EHIS) – Background and methodology

**DOI:** 10.25646/8559

**Published:** 2021-09-15

**Authors:** Jennifer Allen, Sabine Born, Stefan Damerow, Ronny Kuhnert, Johannes Lemcke, Anja Müller, Tim Weihrauch, Matthias Wetzstein

**Affiliations:** Robert Koch Institute, Berlin, Department of Epidemiology and Health Monitoring

**Keywords:** STUDY METHODOLOGY, HEALTH SURVEY, TELEPHONE INTERVIEW, HEALTH MONITORING, EHIS, RESPONSE

## Abstract

Between April 2019 and September 2020, 23,001 people aged 15 or over responded to questions about their health and living conditions for the German Health Update (GEDA 2019/2020-EHIS). The results are representative of the German resident population aged 15 or above. The response rate was 21.6%. The study used a questionnaire based on the third wave of the European Health Interview Survey (EHIS), which was carried out in all EU member states. EHIS consists of four modules on health status, health care provision, health determinants, and socioeconomic variables. The data are collected in a harmonised manner and therefore have a high degree of international comparability. They constitute an important source of information for European health policy and health reporting and are made available by the Statistical Office of the European Union (Eurostat). They also form the basis of the Federal Health Reporting undertaken in Germany. Data collection began in April 2019, just under a year before the beginning of the SARS-CoV-2 pandemic, and continued into its initial phase, as of March 2020. As such, data from the current GEDA wave can also be used to conduct research into the health impact of the SARS-CoV-2 pandemic.

## 1. Background

The German Health Update (GEDA) is conducted regularly by the Robert Koch Institute (RKI) on behalf of the German Federal Ministry of Health (BMG) and is part of the nationwide health monitoring at the RKI [1, 2]. The nationwide telephone survey GEDA 2019/2020-EHIS, the fifth wave of this study, took place between April 2019 and September 2020. The previous cross-sectional surveys were carried out in 2009, 2010, 2012 and 2014/2015, and each involved over 20,000 respondents [3–6].

The aim of the GEDA study is to provide current information about people’s health, the factors that influence their health, and their use of the health care system. The data form an important basis for the Federal Health Reporting (GBE), which provides information about issues relevant to health policy and thus supports policy planning and decision-making processes in Germany. The data are also provided to researchers as a scientific use file.

In its function as national data provider, the RKI also transmits the health data collected in the context of GEDA to the Statistical Office of the European Union (Eurostat). The last wave of the European Health Interview Survey (EHIS) took place in 2019/2020, and was legally binding for all EU member states. The EHIS and its use of statistics are undertaken in line with the European Commission Regulation (EU) 2018/255 of 19 February 2018 implementing Regulation 1338/2008 of the European Parliament and of the Council on community statistics on public health and health and safety at work [7]. The aim of the EHIS is to regularly provide comparable health data from EU member states and, thus, permit analyses of health trends in Europe. Furthermore, the GEDA 2019/2020-EHIS study is aimed at continuing the time series established by health monitoring in Germany. The sample size enables regionalised and deeply structured correlation analyses to be carried out.

**GEDA 2019/2020-EHIS**

Fifth follow-up survey of the German Health Update

**Data holder:** Robert Koch Institute

**Objectives:** Provision of reliable information on the health status, health behaviour and health care of the population living in Germany, with the possibility of European comparisons

**Study design**: Cross-sectional telephone survey

**Population:** German-speaking population aged 15 and older living in private households that can be reached via landline or mobile phone

**Sampling:** Random sample of landline and mobile telephone numbers (dual-frame method) from the ADM sampling system (Arbeitskreis Deutscher Markt- und Sozialforschungsinstitute e.V.)

**Sample size:** 23,001 respondents

**Study period:** April 2019 to September 2020

**GEDA survey waves:**

► GEDA 2009
► GEDA 2010
► GEDA 2012
► GEDA 2014/2015-EHIS
► GEDA 2019/2020-EHIS

Further information in German is available at www.geda-studie.de

## 2. Study design

In accordance with the EHIS regulations, the study population comprises people aged 15 or above living in private households, whose usual residence at the time when the data was collected is Germany. This includes both one- and multi-person households that operate independently and provide for their own needs. As such, collective households such as hospitals, care and residential homes, prisons, military barracks, religious institutions, boarding houses or hostels are not included in the survey. ‘Usual residence’ refers to the place where a person normally lives and views as the centre of their life, irrespective of temporary absences due to recreation, work, medical treatment etc.

The survey used a telephone sample, which was provided by the Arbeitskreis Deutscher Markt- und Sozialforschungsinstitute e.V. (ADM) [8]. It is based on the so-called dual-frame method, in which two selection populations are used: one consisting of mobile phone numbers, and another consisting of landline phone numbers. This sampling method provides (almost) complete coverage of the population in Germany [9]. A method developed by Leslie Kish for the random selection of respondents in multi-person households (the Kish Selection Grid) was used to randomly select prospective respondents [10]. Here, all potential interview partners are given the same selection probability and one person is randomly selected by the computer. This person is identified on the basis of the recorded age and gender.

The interviews began by informing the respondents about the voluntary nature of participation, the survey objectives and data protection; all respondents provided verbal consent to participate. If the target person was unable to conduct the telephone interview, for example due to a cognitive or sensory impairment or due to a long-term absence during the survey, a proxy interview (i.e., another person responds on behalf of the selected person) was refrained from. Some of the topics surveyed in the GEDA study are sensitive and some are highly subjective, so it must be assumed that not all information can be obtained correctly from a proxy respondent.

The data was collected by USUMA GmbH, an external market and social research institute. Staff from the RKI monitored the entire survey process, provided continuous supervision and undertook comprehensive field monitoring (see Chapter 3, Field monitoring).

### Questionnaire

The content of GEDA 2019/2020-EHIS was based on the third wave of the EHIS. As EHIS waves 2 and 3 remained largely unchanged, the data they collected can be used to compare European member states over time. The questionnaire comprised the following four modules:

► Background variables on demographic, geographic and socioeconomic characteristics of participants: including sex, age, education, employment status, country of birth, nationality, marital status, household type and income
► Health status: including self-assessed health, chronic illnesses, accidents and injuries, restrictions to everyday life, disease-specific morbidity, physical and sensory functional limitations, pain and mental health
► Health care provision: including the utilisation of different types of health services (hospital stays, doctor visits, prevention), medicine use, preventive measures and unmet health service needs
► Health determinants: including body mass index (height and weight), diet (consumption of fruit and vegetables), smoking behaviour, alcohol consumption and physical activity

The regulations governing the implementation of the EHIS specify the items to be surveyed including their characteristics and the codes to be transmitted to Eurostat. In addition, the wording of the questions and their response categories, as well as the order in which they are asked, was clarified in a methodological manual and made available in the form of a sample questionnaire (in English) [11]. Compliance with the rules and recommendations was essential to ensure harmonised, high-quality health data could be collected throughout the EU. All EU member states were permitted to add questions to the questionnaire. At this point it should be noted that in GEDA 2019/2020-EHIS an adjustment was made regarding the gender query: in addition to the sex assigned at birth (sex at birth), the gender to which the respondents actually feel they belong (gender identity) was also surveyed. The non-binary question about gender identity enabled the respondents to provide a third open answer in addition to ‘female’ or ‘male’. Respondents 15 years and older included 12,101 women and 10,838 men. 62 respondents indicated a different gender identity (n=28) or gave no information at all (n=34). A detailed description of this procedure will be published elsewhere. With the exception of results based on comparisons with population data taken from the Federal Statistical Office 2019/microcensus 2017, all results reported separately for women and men in this article reflect gender identity. The questionnaire is published as a supplement to this issue of the Journal of Health Monitoring. It can be used for research if the source is provided.

### Survey methods

The most recent GEDA wave was conducted as a telephone interview survey using a computer assisted, fully structured interview (i.e. Computer Assisted Telephone Interview, CATI). The questionnaire was implemented with the help of the ‘VOXCO Interviewer Suite’ software, which offers all the advantages of computer-aided interviews: automated filtering, plausibility checks and defined response areas (range checks). These significantly benefit the quality of the data.

In addition to providing interviewers with a clear graphical interface, the software also offers a complex call management. Telephone number selection, the dialling process and repeated contact attempts are fully automated and undertaken independently of the interviewer.

After programming was completed, the questionnaire routinely underwent several internal quality assurance steps. First, the wording was compared with the programming template in order to detect transmission errors during programming. The questions, the answer categories, and the bridging texts were checked to ensure that they corresponded word for word with the programming template. The functionality of the questionnaire was then examined with a focus on the following areas:

► Branching logic (automatically skipping inapplicable questions),
► Plausibility checks (e.g. error messages if implausible body mass indexes were entered in order to avoid incorrect entries by the interviewer on height and weight),
► Range checks (e.g. error messages if the figures entered were too high or low, in order to avoid incorrect entries),
► Coding of the response categories (mainly supplied by Eurostat).

During testing, particular emphasis was placed on the complex call and callback management built into the questionnaire. Since not every call immediately leads to an interview, all possible call results need to be accounted for in advance so that they can be allocated to disposition codes using the software. Detailed documentation of call results is of crucial importance for the management of callback rules, but it also enables response rates to be calculated. In order to prevent interviewers from inputting incorrect codes, the callback management needs to be effective and easy to use.

In addition to providing effective and detailed documentation of call results, the callback management also fulfils other elementary functions: before the actual interview (survey phase) could take place, interviewees had to be identified and persuaded to participate in the study; this was undertaken during the contact initiation phase. Whereas the survey phase was subject to strict standardisation rules, the callback management system functioned as a guideline for the interviewers during the contact initiation phase so that they could adapt in a tailored and flexible manner to each interviewee. In doing so, the RKI followed the guidelines recommended by the ADM [12]. The extent to which all possible scenarios in the contact initiation phase could be mapped correctly and efficiently via the call and callback management was determined in a pretest (see Chapter 3, Pretesting).

## 3. Survey implementation

### Training approach

An external market and social research institute (USUMA GmbH) was commissioned with carrying out the data collection for GEDA 2019/2020-EHIS. The RKI already has a long-term partnership for the joint implementation of telephone surveys with this institute (GEDA 2012, various ad hoc studies). During data collection, the RKI’s training concept was regularly revised and adapted. The following theoretical units were taught during training sessions (see [13]):

► Information about the client, background and objective of the study,
► Structure, content and special features of the questionnaire,
► Correct technical handling of the CATI software (such as handling disposition codes, navigating the questionnaire),
► Complete, informative, and data protection-compliant documentation of the identification of interviewees and their consent to participate,
► Procedures during the contact phase (interviewing techniques, appropriate conduct),
► Standardised interview management and dealing with information about poor quality interviews,
► Handling difficult situations appropriately (such as digression, pauses in conversation, sensitive questions).

Practical exercises constituted an integral aspect of the training approach and were carried out once the theoretical units had been completed. Among other issues, the interviewers were able to familiarise themselves with the software and practise using disposition codes to code call results with the help of selected example scenarios. Mutual training interviews were extremely valuable, as they enabled the interviewers to adopt the role of interviewees. This provided the interviewers with a feel for the questionnaire’s length, composition and complexity, and enabled them to hone their skills. Moreover, it also helped them to train for difficult calls and thus refine their interviewing techniques.

In addition, a leaflet was made available on the interviewers’ desks summarising all relevant information about the study, key training elements, contact details and how to find more information.

During fieldwork, further interviewers had to be trained to replace interviewers who left the study. As of March 2020, all training courses were carried out online. A total of 216 interviewers were trained during 35 training courses.

### Pretesting

As mentioned in Chapter 2 (Survey methods), the functionality of the questionnaire was tested once programming had been completed. However, some areas of the questionnaire could only be analysed during pretests as they required interviewees. A standard pretest was carried out with a random sample of around 200 interviewees before the survey began. The pretest examined the following aspects and quality criteria (see [14]):

► Comprehensibility: the clarity of the questions was examined in order to ensure that the content and data were being queried and collected as intended (validity)
► Order and logic behind the questions: the order of the question sets was studied to ensure that it was not unconsciously influencing interviewee responses (reliability)
► Filtering: the question sequences were reviewed to make sure that the filters had been programmed correctly (reliability)
► Questionnaire construction and sequencing: the coherence of the questionnaire was examined to avoid unnecessary questions and duplicates (homogeneity and selectivity)
► Call and callback management functionality
► Questionnaire duration: time performance of the overall questionnaire and the question sets

The quality assurance team used the pretest data set to review these aspects and to examine the frequency, distribution of missing values and the length of time required for individual question sets. Feedback was also obtained from the interviewers and supervisors and it was included in the evaluation of the questionnaire.

### Fieldwork

A total of 23,001 interviews were undertaken between April 2019 and the beginning of September 2020. For some regions, the number of interviews was increased to enable the respective federal states to use the data for representative analyses of their own population; in the current GEDA wave, this was done in the case of Berlin and Saarland. Telephone interviews were conducted between Monday and Friday (from 8:30 a.m. to 9:00 p.m.) and on Saturday (from 10:00 a.m. to 3:00 p.m.). They took place in a telephone studio under the supervision of experienced supervisors, and, from mid-March 2020, in line with the measures put in place to contain the SARS-CoV-2 pandemic. Initial contact with potential interviewees usually took place between 2:30 p.m. and 9:00 p.m. On average, 4.3 calls were necessary to complete an interview. The adjusted interview duration was around 40 minutes. There were a total of 216 interviewers – 114 women and 102 men – aged between 19 and 84 years (mean age 53). Diversity was ensured among the interviewers to minimise interviewer effects, i.e. their influence on the responses provided. On average, 1,278 (minimum: 394, maximum: 1,841) people took part in the survey each month.

### Field monitoring

A key aspect of conducting scientific telephone surveys is compliance with a standardised measurement situation (i.e. the interview). To meet this requirement, continuous field monitoring was undertaken and specific criteria were used to continuously monitor quantitative and qualitative aspects of data collection; this enabled specific measures to be derived for field monitoring. The field monitoring undertaken for GEDA 2019/2020-EHIS was based on a standardised concept [15, 16]. Quantitative field monitoring involved observing and evaluating various process data (number of call attempts, interviews, refusals, appointments, average interview duration, etc.). This made it possible to continuously assess the interviewers’ methods and effectiveness and to identify any irregularities in good time so that targeted follow-up training could be offered as early as possible. Qualitative field monitoring was carried out in parallel in the form of supervision. Supervision was conducted by staff from the external market and social research institute and the RKI. During the fieldwork, feedback rounds were held at regular intervals with the interviewers and separate meetings among the supervisors took place where experiences were exchanged. In addition, study-specific information was recorded in a field diary. The supervisors were entrusted with the following tasks:

► Allocation of seating (new interviewers were placed next to experienced interviewers, for example, so that they could learn interview techniques),
► Answering acute questions, such as in dealing with the software or with difficult situations in establishing contacts,
► Quality assurance and contact initiation coaching,
► Quality assurance and coaching of the standardised interview situation.

One of the main objectives of the supervision was to continuously oversee the initial contacts and interviews during the course of data collection and thus to ensure and improve the quality of the work being undertaken. A standardised supervision template (see [16]) was used for this purpose; it was discussed in detail with the interviewers after supervision had been completed and subsequently was archived. These documents were available to supervisors during the fieldwork and formed the basis for the next supervision, so that evaluations could be made of the interviewers’ development over time. If an interviewer had difficulties with contact initiation or (standardised) interviewing, they were provided with follow-up training and, if necessary, additional training in interview techniques. The institutes regularly shared the results gained from these qualitative and quantitative methods. Overall, 1,616 supervisions were carried out during the fieldwork.

## 4. Response

A total of 23,001 complete interviews were conducted (12,620 landline, 10,381 mobile). The response rate (land-line and mobile phone numbers) was determined using the standards of the American Association for Public Opinion Research (AAPOR), whereby the most information-rich result was used instead of merely the last in a particular call sequence [17]. A total of 672,500 phone numbers from the landline sample and 514,823 numbers from the mobile sample were called. As is usual in telephone surveys, most of the numbers were invalid (e.g. unassigned); these were classified using AAPOR codes 4,300 or 4,310 (landline: 524,737 numbers, mobile: 382,044 numbers) [18].

The AAPOR system uses different methods to differentiate between response rates. In simplified terms, phone numbers are assigned to four basic categories depending on the final result: interviews (codes beginning with 1), refus als/non-respondents (codes beginning with 2), unclear phone numbers (codes beginning with 3), invalid phone numbers (codes beginning with 4). Here the Response Rate 3 is reported. Response Rate 3 estimates what proportion of cases of unknown eligibility is actually eligible. It weights phone numbers with an unclear status by providing an estimate of the ‘eligibility rate’; this is calculated as a ratio of refusals/non-respondent calls to invalid numbers. This resulted in a combined response rate (RR3) of 21.6%. The RR3 for the landline sample is 13.8% and 31.0% for the mobile sample. The substantial difference between these rates is mainly due to the much higher proportion of refusals among landline numbers (landline: 8.9%, mobile: 2.7%) and the lower proportion of phone numbers with an unclear status (landline: 3.0%, mobile: 16.9%) in the landline sample.

The need for dual-frame sampling becomes particularly clear when looking at the sample composition differentiated by landline and mobile phone numbers. Although no substantial differences were identified by education, significant differences were found between mobile and landline numbers by gender and age. The mobile sample contains a much larger proportion of 15- to 39-year-olds (30.3%) than the land-line sample (13.2%). In contrast, the landline sample contains a significantly higher proportion of people aged 60 or above (56.3%) than the mobile sample (31.4%). Female participants are also represented much more often in the land-line sample (57.4%) than in the mobile sample (47.1%).

## 5. Data preparation

### Data validation

In addition to the field monitoring measures described above, part of the data quality assurance conducted for GEDA 2019/2020-EHIS involved further extensive checks during data collection. Procedures used to prepare, check and cleanse the data were standardised as far as possible. The methods established for data preparation and quality assurance were supplemented by database tools for the administration and documentation of survey instruments and quality assurance measures. The test procedures developed and specified by Eurostat as part of EHIS were also fully integrated [11].

A reporting tool was used for the first time in the GEDA study during the 2019/2020 wave. This enabled all the relevant information for quality assurance to be displayed clearly and made available centrally to the staff involved in the project. The reporting tool was used for quality assurance throughout fieldwork so that errors in the data collection process could be identified and action could be taken immediately. Compliance with EHIS specifications (consistency checks, filters) was also reviewed when the data was being cleansed and prepared. The reporting tool was updated monthly.

Data cleansing and quality assurance specifically involved checking that the correct form of filtering was being implemented, identifying and correcting implausible information (e.g. value ranges, inconsistencies), and generating new variables. The guidelines developed by Eurostat were also followed during this process. This led to the implementation of three different groups of rules: code reviewing and value ranges (value check, VC), filter checks (skip check, SC) and checking the plausibility between different sub-topics (consistency check, CC). In addition, free text coding and income imputation (replacement of missing income information with statistical methods) were also carried out.

Since the study used computer-assisted telephone interviews (CATI), aspects of filtering and plausibility checks could already be incorporated during the construction of the survey instrument. For example, filters were built in during programming to implement the skip checks specified by Eurostat, so that filter violations could largely be ruled out. Value checks were also built into the questionnaires to ensure that values were within plausible ranges, which is why only a few details (e.g. on income and household composition) had to be examined in more detail afterwards and set to missing in some cases.

These types of checks may also require the data to be further reviewed. However, although warnings may highlight certain values as implausible, they may actually be valid. In contrast, error messages may mean that values have to be replaced with valid input.

The Indicators Manual provided by Eurostat contained a list of the variables to be generated in order to be able to perform international comparisons, for example with previous EHIS waves or between EU countries. The RKI’s Epidemiological Data Centre generated the required variables centrally for the evaluation data set and a detailed data information was created.

Sometimes open answers had to be inputted, which was the case with ‘professional qualifications/occupation’ and, to a lesser extent, gender identity. The responses on gender identity were evaluated by experts and the responses were assigned the appropriate codes. The responses to the questions about occupation were initially coded using the (national) Classification of Occupations 2010 (KldB10) [19, 20]. This involved computer-supported manual coding using software programmed and developed at the RKI. After the data had been recorded using KldB10 codes, the codes were changed to those used by the International Standard Classification of Occupations 2008 (ISCO 08) [21]. The majority of these codes were automatically converted using a unique conversion key. The remaining codes – approximately 30% – were assigned manually. The maximum number of ISCO-08 codes is four.

### Weighting

The weights indicate how many people from the general population are represented by one person in the sample. Weighting typically involves design and adjustment weighting. The design weights are determined by the probability of a particular person being selected for the study (selection probability). People with a lower selection probability represent more people from the population than people with a high probability of selection. As discussed in Chapter 2, the sample was based on a combination of mobile and landline numbers. The resulting design weights are based on a standard calculation method used for the dual-frame design presented here [22]. The calculation was conducted by the market and social research institute commissioned with carrying out the survey.

Adjustment weighting aims to balance out possible differences in willingness to participate in the study. If people from certain population groups are less willing to take part in a study, they will be less represented in the sample than in the actual population. The sample was adjusted to account for potential bias using population data supplied by the Federal Statistical Office (Destatis) and the micro-census 2017. The population was divided into non-overlapping subpopulations (strata) for which the population numbers are known. In the sample, the weights were adjusted in each stratum to ensure that the figures correspond to the external information. In order to do so, the sample was divided by federal state, residential structure [23], age, sex and education (in line with the International Standard Classification of Education, ISCED11 [24]). Information on sex at birth was used so ensure that the sample could be compared with the population projection. Adjustment weighting was carried out iteratively using raking [25]. This procedure was repeated until very little change was noted between the figures. After each adjustment stage, weights that were lower than the 0.5% quantile or greater than the 99.5% quantile were set to the value of the nearest quantile. For evaluations of sub-samples with participants aged 18 or over, an extra weighting factor was applied, which was established using the same procedure. During weighting, it is imperative that all relevant variables have valid values. Missing values were therefore replaced by valid values (most common category in education; imputation of state information and district type).

Overall, the unweighted proportions by age group and sex show a relatively good correlation with the weighted proportions that correspond to official population figures. There are certain differences between respondents under 45 years of age, who are underrepresented in the unweighted sample (Table 2), and respondents between 45 and 79, who are over-represented. Table 2 demonstrates that fewer people in the low education group were prepared to be interviewed; on the other hand, there was a greater willingness to participate among the high education group. This educational bias in the sample was also identified by the GEDA 2012 study [5].

## 6. Strengths and Limitations

The integration of EHIS into the GEDA 2019/2020-EHIS study makes it possible to compare health data from Germany with relevant data from EU member states and to conduct analyses at the European level. However, it should be noted that survey modes and sample designs vary between countries and this must be taken into account when evaluating results [26].

Trend analyses can be conducted for certain aspects using data from previous GEDA waves (2009, 2010, 2012) as these were also conducted as telephone-based studies. The data from the GEDA 2014/2015-EHIS survey is comparable with the current wave as the questionnaire has remained largely unchanged. However, the sample design was changed from a register sample to a telephone sample, which is why conclusions about trends are only possible with restrictions. In addition, the survey mode altered from a self-administered questionnaire (online, paper) to a computer-assisted telephone interview (CATI). Results indicating breaks in health-indicator trends in Germany and, therefore, need to be treated with caution. However, a methodological study found that study mode had very little impact on the prevalence of some health indicators, although it was shown to affect others more strongly [27]. As data collection was undertaken between April 2019 and September 2020, EHIS partly took place during the initial phase of the SARS-CoV-2 pandemic [28]. In addition to aspects of COVID-19, the measures put in place to contain the pandemic had an impact on many other aspects of population health. These measures may also have influenced people’s willingness to participate in the study. The expansion of flexible working from home and the increased use of short-time work could mean that certain population groups were easier or more difficult to reach by telephone. Such impacts have already been observed in the literature. A study from the United States, for example, found that willingness to participate in the 2020 census significantly reduced in line with increasing infection rates (postal recruitment) [29]. When analysing the data from GEDA 2019/2020-EHIS, the potential impact of the pandemic on health and possible changes in willingness to participate need to be considered. This can be done using sensitivity analyses and, if necessary, these impacts can be accounted for, for example, by correcting weighting factors. Despite these limitations, GEDA 2019/2020-EHIS provides unique data for research into the health impact of the SARS-CoV-2 pandemic (see [30] and the Fact sheet Utilisation of outpatient medical services by people with diagnosed diabetes during the COVID-19 pandemic in Germany in issue 2/2021 of the Journal of Health Monitoring). EHIS is representative of the population in Germany and uses consistent survey instruments to depict temporal developments. No other survey was found that does this while also enabling direct comparisons to be made about population health around a year before the outbreak of SARS-CoV-2 pandemic with the period that immediately followed (March 2020). It is important to note that the selection framework used for the ADM sample is an established research tool. It enables high-quality random samples to be drawn from the general population. In addition, the use of a telephone interview means that a fully standardised survey mode was selected that can be used efficiently and relatively quickly. Potential interviewer effects (cluster effects) are less pronounced with telephone interviews than with face-to-face surveys [31]. At the same time, telephone interviews provide the possibility of conducting efficient quality assurance by continuously supervising the interviewers [32]. However, these surveys also have limitations compared with other survey modes. Like all interviewer-based surveys, telephone interviews are prone to socially desirable responses. In the case of potentially sensitive questions, this can lead to underestimates of ‘true’ prevalences [32]. Finally, reported response rates for telephone surveys are generally lower than for face-to-face interviews and this can increase the risk of non-response bias, although a low response rate need not automatically lead to biased results [33].

## Key statements

- GEDA 2019/2020-EHIS is a cross-sectional telephone-based study of the population in Germany in which 23,001 people provided information about their health.
- The data are used for Federal Health Reporting in Germany. The Statistical Office of the European Union (Eurostat) uses them to compile official European statistics.
- As part of EHIS, EU member states collect data every six years on the health status, health care provision and health determinants of the population aged 15 and older.
- GEDA data enable comparative analyses to be conducted at European level.
- GEDA is the largest population-based health survey of adults in Germany and involves more than 20,000 respondents per wave.

## Figures and Tables

**Table 1:** Response by sociodemographic characteristics, broken down into landline and mobile phone numbers Source: GEDA 2019/2020-EHIS

Characteristic	Landline		Mobile	
	n	*%*	n	*%*
**Gender (gender identity)**				
Female	7,227	57.4	4,874	47.1
Male	5,359	42.6	5,479	52.9
**Age group**				
15–39 years	1,665	13.2	3,145	30.3
40–59 years	3,852	30.5	3,974	38.3
≥60 years	7,103	56.3	3,262	31.4
**Education level (ISCED classification 2011)**				
Low education group	946	7.5	673	6.5
Medium education group (No A-Levels)	3,864	30.7	2,701	26.1
Medium education group (A-Levels)	1,567	12.5	1,550	15.0
High education group	6,212	49.3	5,425	52.4

ISCED=International Standard Classification of Education

**Table 2:** Description of the sample by sociodemographic characteristics and total number, unweighted, weighted and compared with population data from the Federal Statistical Office 2019/microcensus 2017 Source: GEDA 2019/2020-EHIS

Characteristic			Weighted	Destatis 2019/Microcensus 2017^**^
	n	*%*	*%*	*%*
**Sex (biological sex)**				
Female	12,111	52.7	51.0	51.0
Male	10,890	47.3	49.0	49.0
**Age group**				
15–29 years	2,394	10.4	18.9	19.0
30–44 years	3,769	16.4	21.9	21.9
45–64 years	8,981	39.1	34.0	34.0
65–79 years	6,048	26.3	17.4	17.3
≥80 years	1,809	7.9	7.8	7.9
**Residential structure of district (BBSR)**				
Sparsely populated rural areas	2,554	11.9	14.9	14.9
Rural districts	2,830	13.2	17.1	17.1
Urban districts	8,385	39.1	37.8	38.5
District-free cities	7,664	35.8	30.2	29.4
Education level* (ISCED classification 2011)				
Low education group	1,339	5.9	17.8	17.5
Medium education group (no A-Levels)	6,560	29.0	41.9	42.2
Medium education group (A-Levels)	3,109	13.7	15.1	15.2
High education group	11,637	51.4	25.1	25.2

BBSR=Federal Institute for Research on Building, Urban Affairs and Spatial Development, ISCED=International Standard Classification of Education

^*^ Only participants aged 18 or over are shown, as a large proportion of the population aged 15 to 17 has yet to complete an education level described by ISCED11

^**^ Sex, age and residential structure based on population data from the Federal Statistical Office 2019; ISCED education groups based on the 2017 microcensus
